# Multi-Organism Composites: Combined Growth Potential of Mycelium and Bacterial Cellulose

**DOI:** 10.3390/biomimetics7020055

**Published:** 2022-05-03

**Authors:** Aileen Hoenerloh, Dilan Ozkan, Jane Scott

**Affiliations:** Hub for Biotechnology in the Built Environment, School of Architecture, Planning and Landscape, Newcastle University, Newcastle upon Tyne NE1 7RU, UK; d.ozkan2@ncl.ac.uk (D.O.); jane.scott@newcastle.ac.uk (J.S.)

**Keywords:** mycelium, bacterial cellulose, biocompatibility, knitted fabric, material tinkering

## Abstract

The demand for sustainable materials derived from renewable resources has led to significant research exploring the performance and functionality of biomaterials such as mycelium and bacterial cellulose. Whilst the growing conditions and performance of individual biomaterials are understood, to achieve additional new and enhanced functionality, an understanding of how biomaterials can be used together as composites and hybrids is required. This paper investigates the compatibility of mycelium and bacterial cellulose as two biomaterials with different qualities for the development of a large-scale biohybrid structure, the *BioKnit* prototype. Their compatibility was tested through preliminary design experiments and a material tinkering approach. The findings demonstrate that under optimal conditions mycelium and bacterial cellulose can grow in each other’s presence and create composites with an extensive array of functions. However, there is a need to develop further fabrication settings that help to maintain optimal growing conditions and nutrition levels, whilst eliminating problems such as contamination and competition during growth.

## 1. Introduction

The growing interest in the use of living materials in the design field is motivated by the desire to create a renewable resource of sustainable materials that is biodegradable and grown using waste materials. This approach offers the potential to produce a low-cost alternative to commercial synthetic materials whilst developing a new spectrum of functional properties [1]. For instance, while fungi as mycelium composites can be used as a structural bulk material as in the MycoTree, MycoCreate-2, and El Monolito Micelio projects [2,3,4,5], bacterial cellulose shares many properties with plant cellulose and is already successfully used in the medical field [6] and in fashion design projects [7,8].

Whilst current research has focused on optimising the growth and performance of individual microorganisms for design applications, our research considers the potential to combine different microbial systems in order to achieve new functional possibilities. For example, can multi-microbial systems produce stronger, more durable biomaterials whilst transforming the look and feel of materials for architecture? Using biological materials at different stages of their lifecycle for different purposes requires developing new methods of fabrication that allow the designers to reveal their various features. *BioKnit* is a prototype designed and built in the Hub for Biotechnology in the Built Environment (HBBE) under the Living Construction theme [9]. It focuses on bringing mycelium and bacterial cellulose together with textiles using knitting technologies. Knitted fabric, the first component of the prototype, acts as a scaffold and guides, enhances, and restricts the organism as it grows or attaches to it. Mycelium in a composite form, the second component, acts as a bulk material that gives sufficient compressive strength to the knitted structure to enable the production of a 1.8 m high, free-standing vault. Bacterial cellulose (BC) is the third component and adds a layer of complexity to the system by adding a new optical and tactile quality. BC is used as a surface treatment to coat the knit/mycelium composite and as a tactile skin, self-adhered to the mycelium/knit composite. Mycelium and BC, as two commonly used biomaterials in the design field, were chosen as organisms since cellulose can be used to cultivate mycelium [9]. It proves that they can establish a relationship, and one of the organisms could be used to support the other.

Fungi mainly consist of two parts: the fruiting body/mushroom and the mycelium/roots. The mycelium is of general interest as a biomaterial due to its ability to rapidly grow on various forms of waste as a composite, creating a bulk building material [5]. There are numerous studies on the properties of mycelium composite materials grown on agricultural waste, such as the works of Appels et al. [10] and Jones et al. [5]. Another approach was taken by Elsacker et al. by growing BC to be utilized as a nutritious substrate additive with the focus being on improving the mycelium growth and incorporating the bio-organism in a dried form [11].

BC is a biopolymer that can be grown as a pure culture from a single bacterial strain in sterile lab conditions or with a symbiotic culture of bacteria and yeast (SCOBY), which is commonly used to create fermented kombucha tea in household kitchens. Properties such as fine crystalline structures, biodegradability and biocompatibility, good water-holding capacity, and chemical stability can explain the growing interest in BC as a biomaterial [6]. Due to the large-scale nature of the *BioKnit* prototype, the more resilient kombucha method was chosen for these experiments.

This paper introduces the initial design experiments that were conducted during the prototyping process and asks the following questions: (1) how two organisms grow together and how they influence each other, (2) what is the potential of a knit scaffold as a technique to assemble multiple living materials through growth, and (3) how can the challenge of contamination due to varying growth requirements be addressed? These questions are tested to develop protocols for multi-kingdom textile composites through a methodology based on explorative experimentation using material tinkering [12] and based within established biomaterial protocols rather than a biomimetic investigation translating functional models from nature. Each organism’s behavior was observed in an iterative array of experiments.

## 2. Materials and Methods

### 2.1. Mycelium Composite Preparation

Mycelium grown in the experiments was inoculated with a mixed substrate (10 g of strawbale, 10 g of wood shavings, 10 g of coffee grounds), which was sterilized in an autoclave at 121 °C for 15 min. The sterile mixture was seeded with 10 g of oyster mushroom spawn from GroCycle, UK and kept in sealed plastic boxes (100 × 100 × 30 mm), in the dark, at ambient temperature. After three weeks, the samples were taken out of the boxes and kept in three different forms: oven-dried, air-dried, and living. The first set was air-dried for 8 days and then oven-dried (for 2 h under 60 °C). The second set was air-dried for 2 weeks at room temperature. Finally, the third set of tiles were kept alive in a closed container. These three sets of tiles were then integrated in the bacterial cellulose experiments. 

### 2.2. Bacterial Cellulose Preparation

All BC was grown using the kombucha technique with a tea-based medium and a piece of SCOBY (symbiotic culture of bacteria and yeast) purchased from “Happy Kombucha”. A new SCOBY was used for each experiment. The medium was prepared in 3-litre batches with tap water, pasteurized apple cider vinegar (62.5 mL/L), white refined sugar (75 g/L), and black tea (2 g/L) (Tetley Black Tea bags). To prepare the medium, the water was brought to boiling point before adding the tea bags to infuse for 15 min. The vinegar and sugar were added, then stirred until all the sugar had dissolved. The addition of vinegar lowers the pH from 4.9 to 3.5 (±0.1). The medium was cooled down to <30 °C before being used. The BC in experiments 1–3 was grown in a plastic box (110 × 190 × 330 mm) with 2.2 L medium, covered with a cotton cloth. Experiment 4 was grown in a (360 × 26 × 140 mm) box with the medium quantity adjusted throughout.

### 2.3. The Experimental Setting

The associations of BC and mycelium in different metabolic stages were tested in four experiments. Mycelium composites were shaped as tiles, and BC was oriented flat due to the necessity in its growth conditions. 

The experiments were documented using photography (iPhone SE and Fuji film X-T2 with 80 mm lens) and microscopy (Dino-Lite digital microscope at 70× magnification). The images from photography and microscopy captured the details, smaller features, and non-measurable characteristics such as the surface texture and BC attachment. They generated qualitative data for this study.

In experiments 1, 2, and 3, the compatibility of BC (secondary organism) growing around already-established mycelium (primary organism) was explored. In experiment 4, the compatibility of mycelium (secondary organism) growing around already-established BC (primary organism) was explored. In each experiment, the success of the growth of the secondary organism was monitored alongside the contamination that occurred during the growth stage.

### 2.4. Experiment 1 (Oven-Dried Mycelium with BC)

**Figure 1:**
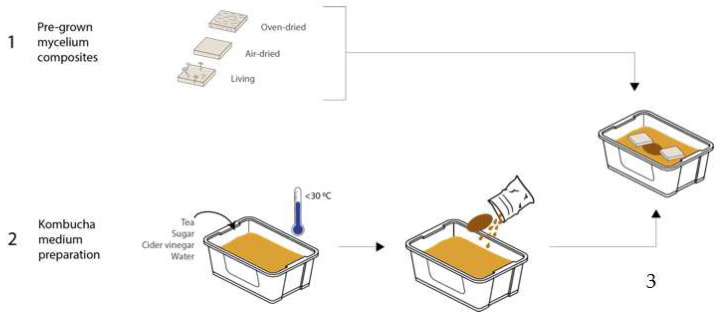
The setup for experiments 1, 2, and 3 (image credit: authors).

After preparing the kombucha culture, a fresh SCOBY was placed in the medium, as seen in Figure 1. Then, the first set of oven-dried mycelium tiles were positioned on the surface. It was not necessary to add support and keep the mycelium at the liquid surface, as the hydrophobicity of it kept the tiles afloat. The SCOBY was positioned in between the tiles towards the center of the container. This setup was left in a static condition to ferment for 14 days.

To harvest the composite material of BC and mycelium of experiments 1–3, the growth was removed from the liquid medium and washed with antibacterial dish soap and cold water after 14 days of static fermenting. While the BC was thoroughly kneaded with the soap, care was taken to not wet the mycelium or introduce stress to the joint locations of the BC to the tiles.

### 2.5. Experiment 2–3 (Air-Dried Mycelium with BC and Living Mycelium with BC)

**Figure 2:**
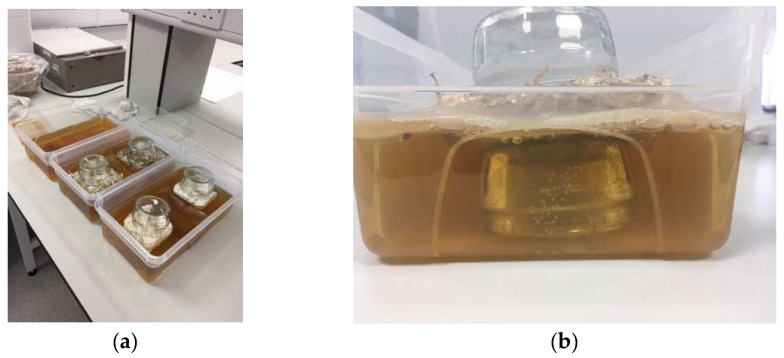
(**a**) Setup of experiments 2 and 3 with (from right to left) air-dried mycelium, living mycelium, and a control culture. (**b**) Closeup of the positioning of the tiles within the liquid using jars (image credit: authors).

For experiments 2 and 3, the hydrophobicity of the air-dried and living mycelium was reduced and not sufficient to keep the tiles afloat; therefore, the placement of the tiles in the liquid medium was adjusted. Two small glass jars were used to support each tile: one upside down and submerged in the liquid to place the tile on (see Figure 2) and the other one placed on top to weigh down the otherwise floating tile. The jar was placed with the opening facing down to allow ventilation and avoid the growth of mold underneath. The quantity of medium was adjusted to cover the bottom half of the tiles, and the SCOBY was placed in between the tiles in the center of the box.

The harvesting process for these experiments was the same as for experiment 1.

### 2.6. Experiment 4 (BC with Living Mycelium inside Soft Scaffold)

**Figure 3:**
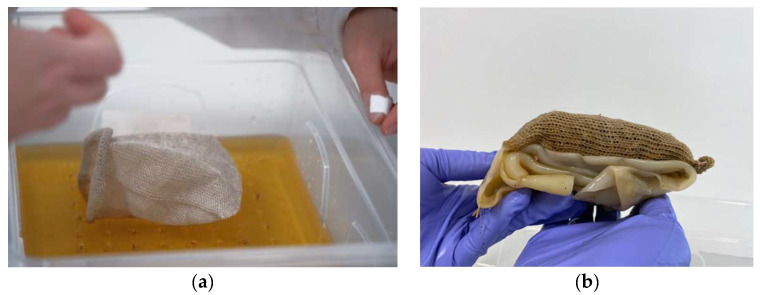
(**a**) The knitted pocket being placed inside the kombucha culture on top of the acrylic scaffold with the top half being lifted. (**b**) Pocket filled with pre-inoculated mycelium substrate and BC folded in 9 layers underneath (image credit: authors).

In experiments 1–3, the compatibility of BC growing around already-established mycelium was explored; however, it was also important to understand whether the order of growth could be reversed, with mycelium growing as a secondary organism alongside already-established BC. This was tested in experiment 4. For the mycelium to grow next to or inside wet bacterial cellulose, a support that can hold and contain the substrate is needed. In line with the *BioKnit* background of this research, a knitted pocket (linen, circular plain, 8 gg Dubied) was used to contain the mycelium substrate. To avoid killing the bacteria in the BC, the mycelium substrate was autoclaved and pre-inoculated for 5–10 days. Simultaneously, the BC was grown on the soft scaffold. To stop the BC from merging both sides of the pocket together, one side was placed on top of an acrylic scaffold inside the kombucha medium to be held on the air–liquid interface while the other side was pulled up on the corners with acrylic string (see Figure 3a). Fresh medium was added underneath the growing pellicle every 3 days to compensate the evaporated medium and keep the air–liquid interface stable.

**Figure 4:**
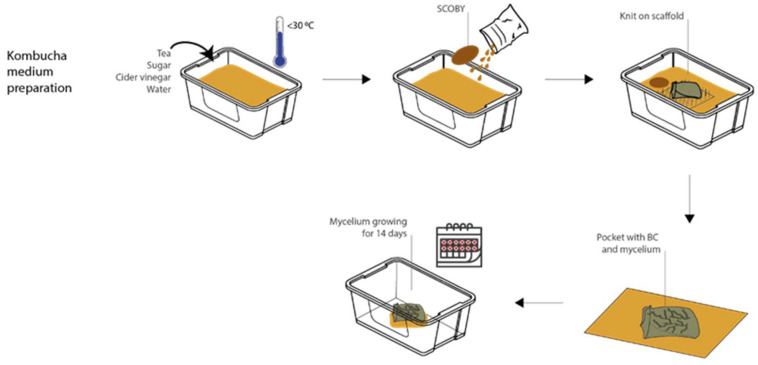
The setup for experiment 4 (image credit: authors).

The second step of the experiment involved harvesting the BC knit composite and filling the pocket with the pre-inoculated mycelium substrate (see Figure 3b and Figure 4). The pocket was sewn shut using cotton string, piercing through the layer of BC and the knit. To allow the mycelium to breathe, the excess BC was folded underneath the pocket on the side which had the BC growth on it (total of 9 BC layers), leaving one knitted side exposed (Figure 3b). This composite was placed inside a plastic box with lid, BC side facing down, and left to grow for 12 days.

Experiment 4 required two separate harvesting processes. First, the BC was harvested after 14 days of static growth and washed as described above. The top side of the knitted pocket was also cleaned with dish soap to remove any media that had been drawn up through the fibers. The fabric was thoroughly rinsed to eliminate the risk of soap residue hindering the mycelium growth. The second harvest stage was after 12 days of mycelium growth, where the composite material was removed from the plastic box and dried. Because contamination occurred, the composite was dried in an industrial oven at 50 °C for 8 h.

Throughout the 14 days of BC growth, the formation of air bubbles was handled as in the previous experiments and pushed out to the sides.

### 2.7. Drying Process

The drying process for experiment 4 is described above. For experiments 1–2, the BC was washed, padded dry using paper towels, and placed on a clean bamboo cutting to dry uncovered at 21 °C. The composite was placed with the BC side on the tiles facing down. After 48 h, the composite was turned over, exposing the BC on the tiles. After further 48 h, the BC had turned translucent and fully dried. For experiment 3, the samples that showed signs of mold contamination were not dried on a bamboo board but dried in an industrial oven at 60 °C for 4–6 h.

## 3. Results

### 3.1. Results of Experiment 1 (Oven-Dried Mycelium with BC)

**Figure 5:**
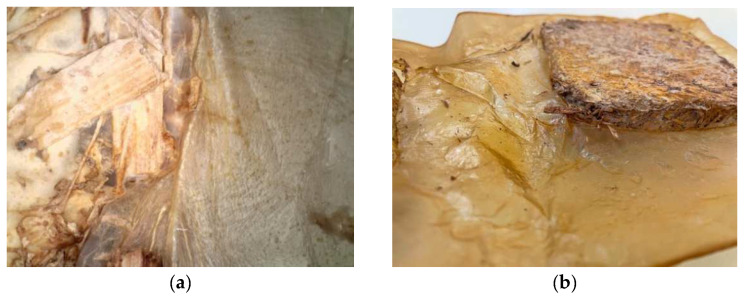
(**a**) Dried BC around oven-dried mycelium tile under the Dino-Lite digital microscope. (**b**) Large BC sheet dried around mycelium tile (image credit: authors).

In experiment one, good BC growth occurred; however, the resultant growth was uneven across the surface. Growth was measured on day 14. The BC thickness ranged from 12 mm at the outer edges of the cellulose to 2 mm around the SCOBY (where a bubble had formed). The thicker areas of the pellicle showed good connection to the mycelium tile and did not detach while handling; however, the 2 mm BC ripped during the harvesting process. After drying, the tiles were firmly integrated into the pellicle, and the composite could be held up from the tiles without the cellulose detaching or ripping (Figure 5).

The oven-dried tiles were very hydrophobic and initially moved freely within the liquid medium. On day three of the fermentation, a thin and clear layer of cellulose had grown on the surface, which stopped the mycelium tiles from moving around. In the center of the pellicle, a large bubble of gas built up where the SCOBY had been positioned during the fermentation. This bubble lifted the cellulose off the liquid and stopped the growth of the cellulose at this area after less than 7 days. One of the tiles was also lifted into an angled position. 

The formation of CO_2_ during the fermentation is a normal occurrence during the kombucha method; however, the visible amount of trapped CO_2_ produced in this experiment was greater than in a control setup without mycelium present. The gas bubble was problematic because it limited BC growth in particular areas and disturbed the position of one of the mycelium tiles. This led to a poor connection between the BC and mycelium close to the gas bubble. 

### 3.2. Results of Experiment 2 (Air-Dried Mycelium with BC)

In experiment two, good BC growth occurred producing a more even pellicle after 14 days of growth. Growth was measured on day six and day 14. On day six, there was a visible layer of cellulose on the surface that measured 2–3 mm. By day 14, growth measured between 10–13 mm across the whole surface. During the growing stage, there was a very weak attachment between the mycelium and the BC. This was clear during harvest because the BC fully detached from the mycelium. Despite this, during drying the BC adhered to the mycelium. The attachment was maintained even with heavy handling.

**Figure 6:**
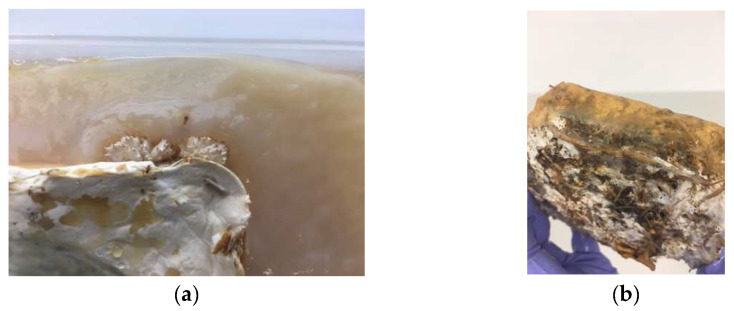
(**a**) Thicker BC growth around dried fruiting body. (**b**) BC skin around submerged half of mycelium tile after harvest (image credit: authors).

During BC growth, many small bubbles formed underneath the pellicle. To maintain growth, the bubbles were gently pushed to the side of the pellicle using sterile hands. This was continued each day until day 10, when the stiffness of the pellicle prevented intervention. Cellulose growth was thicker around dried fruiting bodies (the side of the mycelium tiles). This formed a patchy appearance overall (Figure 6a). In addition, the BC had formed a skin-like film under the submerged half of the tile, even without direct access to oxygen (Figure 6b). This indicates that the mycelium can provide a small amount of oxygen to the bacteria.

### 3.3. Results of Experiment 3 (Living Mycelium with BC)

In experiment three, BC growth was changed by the presence of a living mycelium. Growth was measured on day six and day 14. On day six, the pellicle thickness was 2–3 mm in line with previous results. However, by day 14 the pellicle had only grown to a thickness of between 3–5 mm. BC growth was observed across the mycelium tile in gaps within the material, and this led to good attachment during the growing stage. Due to contamination, this sample was oven-dried, and this drying process also created the strongest connection between the mycelium and the BC.

Aeration bubbles again appeared underneath the pellicle; however, these were much smaller and greater in number, leaving a foam-like appearance. Larger bubbles also formed, and these were pushed to the side. The living mycelium tiles had fruiting bodies that grew out on the side and were either hovering just above or within the air–liquid interface of the medium. The BC grew around the fruiting bodies and enclosed them. The mushrooms sitting half-submerged on the surface began to dry out and had shrunk in size by day seven, but the connection to the BC remained. Unlike the mycelium tiles in experiments one and two, these tiles were not hydrophobic, and they soaked up the liquid medium. During the experiment, the top of the tiles also became submerged. Where the top of the tile soaked up the medium, some BC growth occurred. Around the side of the tiles, the BC growth was uneven, with thinner parts forming the connection to the mycelium (2–3 mm) and the outside edges and corners being the thickest parts. As in the previous experiment, the cellulose growing around bunches of primordia formations was thicker. During the first 7 days, the area of the tile that was covered with the upside-down jar continued to grow white patchy mycelium. This growth slowed down and eventually stopped once the jars were removed.

Contamination in the form of green mold occurred on the top surface of one of the tiles where the medium had been soaked up. This meant that the composite could not be air-dried after harvest but needed to be oven-dried at 100 °C for 1 h. Compared to the air-dried versions, the BC was darker in color and significantly more brittle. During the harvest, the tiles soaked up the washing water and needed to be handled very carefully to not fall apart. The BC did not hold the tile substrate in shape.

**Figure 7:**
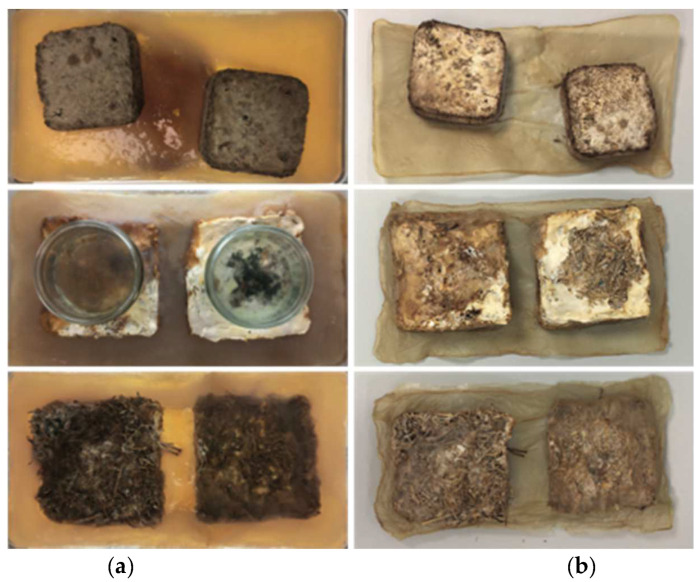
Comparison of experiments 1–3 in the order oven-dried, air-dried, and living from top to bottom in (**a**) wet state and (**b**) dried state (image credit: authors).

Figure 7 shows a visual comparison of experiments one, two, and three in a hydrated and dried state.

### 3.4. Results of Experiment 4 (BC with Living Mycelium inside Soft Scaffold)

**Figure 8:**
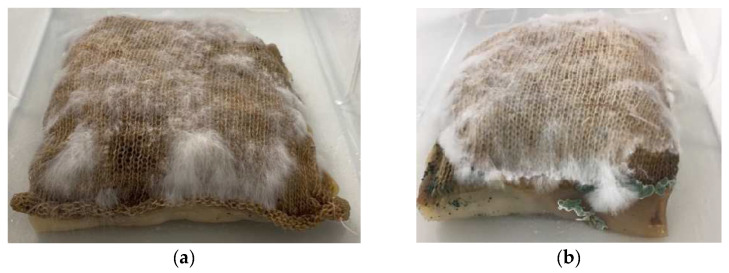
(**a**) Mycelium growth on the knitted pocket on day 7. (**b**) Mycelium growth on the knitted pocket on day 14 (image credit: authors).

In experiment four, mycelium growth was contained in a knitted pocket submerged in BC. The results showed that the mycelium grew alongside the BC; however, mycelium growth was uneven (in comparison to controls with no BC present). In this experiment, the attachment between the mycelium and BC was created by the knitted pocket, and this was effective in bringing the two organisms together. After 12 days, the BC showed signs of contamination in the form of mold; therefore, the composite was oven-dried (see Figure 8). The connection between the mycelium and BC as a dried composite was strong, with the texture of the fabric clearly visible through the BC.

The first stage of the experiment resulted in an even growth of BC (5 mm) attached to the bottom half of the knitted pocket. Due to the high absorbency of the fabric used to create the pocket, the starter liquid for the kombucha culture partly saturated the top layer, which was suspended over the air–liquid interface. No visible layer of the BC formed on those parts of the pocket. 

During the second stage of the experiment, the growth behavior of the mycelium was observed. In the first 7 days, the organism started to visibly grow around the sides of the pocket, as seen in Figure 7. The larger patches of mycelial growth were located predominantly along the edges due to the level of moisture on those parts. However, the knitted pocked was not fully covered by a dense layer of mycelium. This was possibly due to the linen being difficult to digest for the mycelium. The BC had not begun drying at this time. The fabric on the top side had also not fully dried from the washing. 

After a total of 12 days, the wet BC showed signs of contamination in the form of mold. The mycelium had continued to grow along the edges of the pocket and onto the plastic container. The very top of the fabric showed less growth than before. The corners of the pocket, where the liquid medium had been soaked up during the first stage, showed no mycelium growth but contamination reaching from the BC. Those areas of the fabric showed dark discoloration.

**Figure 9:**
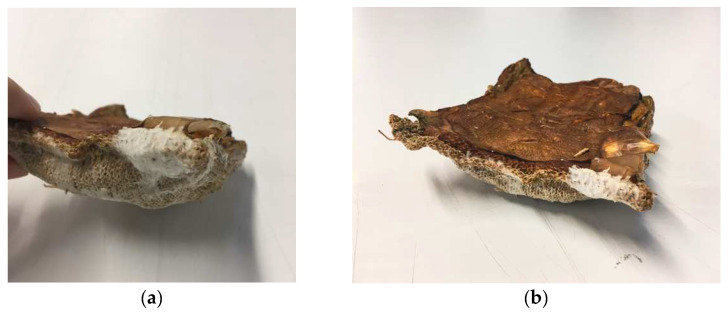
(**a**) Corner of the pocket with white mycelium skin. (**b**) BC dried onto the pocket, showing pattern of fabric underneath (image credit: authors).

After drying the composite in an oven, the mycelium and BC both significantly decreased in thickness. Only one corner of the pocket showed signs of a beginning white coating (see Figure 9a), as have been observed in prior experiments. While the BC was very brittle, it showed strong attachment to the fabric and had molded onto it in a way that the texture of the fabric came through.

## 4. Discussion

The primary purpose of this study was to test the compatibility of the two chosen biomaterials and identify methods of growing them into viable composites. The results of all four experiments demonstrated that reliable growth of a secondary organism was achievable in the presence of a primary organism (Table 1). However, we were not able to avoid contamination in the wet stage of the growth process in experiments three and four when both the mycelium and BC were alive. Although pre-grown mycelium composites are less receptive to contamination, the increased humidity and presence of sugar from the BC culture created a beneficial environment for mold, which was observed in experiment three. This problem could be solved by either adjusting the timeline of the setup, allowing the BC to dry partly before adding the mycelium in experiment four, or by adding active ventilation underneath the BC inside the mycelium growth box. For experiments 1–3, a more refined method to place the tiles inside the kombucha medium can be designed to maximize free air flow on the surface of the mycelium. Whilst contamination is evident in experiments three and four, it is notable that this has not prevented successful attachment between the mycelium and the bacterial cellulose; in fact, the best attachment was observed in experiment four, where mold was observed on both the BC and mycelium.

The presence of mycelium in any state of aliveness caused greater production of fermentation gases, which led to visibly thinner BC in the areas where gas collected in bubbles. The BC also showed increased growth around fruiting bodies and primordia formations, indicating a greater availability of nutrients and/or oxygen in these areas through the mycelium composite. 

The shape of knit in the form of a pocket was valuable as a scaffold for the BC to grow around and to hold the loose mycelium substrate in a compacted shape. The knit successfully offered a link between the two organisms in the setup. The growth behavior of mycelium with different types of yarn was tested in a separate set of experiments as part of the *BioKnit* project, which revealed linen to be less compatible [13]. This can partly explain the limited mycelium growth in experiment four.

## 5. Conclusions

This paper focused on the biological compatibility of mycelium and bacterial cellulose as two biomaterials that produce different and potentially complimentary qualities for a new generation of biohybrid materials for architecture. Through experimental study, the research investigated four different combined growth setups to test the potential for growing together, the use of a knit scaffold, and the challenge of contamination.

Growing Together: The research demonstrates that BC and mycelium can grow effectively in the presence of one another. The growth of the secondary organism is influenced by the growth requirements of the primary organism, particularly when both organisms are living. This influence was observed predominantly through uneven growth of the secondary organism. In two experiments, the presence of mushrooms growing from the mycelium increased the BC formation in the surrounding area, suggesting a localized increase in nutrition supply for the BC. 

The use of a knit scaffold: Whilst BC and mycelium can grow together, producing a strong attachment during growth, forming a composite is more problematic. The knitted scaffold proved helpful in bringing the two organisms together in a way that supported both growth behaviors through the fabric’s adjustable breathability and rough texture. As a result of this research, large-scale knit scaffolds have been implemented in the design of the *BioKnit* prototype. Further research investigating attachment strategies could guide the design of future textile scaffolds.

The challenge of contamination: The changing environmental conditions required by the different growth requirements of BC and mycelium increase the probability of contamination during the growth phase. The most challenging factors observed are humidity and ventilation leading to the growth of mold. Further research is required to optimize the environmental conditions, and active systems to control ventilation and humidity during growth will be developed to eliminate these problems in future projects.

## Figures and Tables

**Table 1:** The summary of the experiment.

	Oven-Dried (No. 1)	Air-Dried (No. 2)	Living (No. 3)	Pocket (No. 4)
BC growth	5–13 mm, <2 mm on gas bubble	5–8 mm	5–8 mm	4–6 mm and inclusion of knit
Mycelium growth	Dead, white film around substrate	Dormant pinheads, white film	Mushrooms and no white film	Little and on top side only
Attachment	Good	Weak to not at all	Stronger after oven drying	Strong
Contamination	No contamination	No contamination	Mold on exposed mycelium	Mold on BC and mycelium
Other observations	Uneven BC growth	BC around fruiting bodies thicker	BC grew around mushroom	-

## Data Availability

Not applicable.
